# Hierarchical Porous Carbon Electrodes with Sponge-Like Edge Structures for the Sensitive Electrochemical Detection of Heavy Metals

**DOI:** 10.3390/s21041346

**Published:** 2021-02-14

**Authors:** Jongmin Lee, Soosung Kim, Heungjoo Shin

**Affiliations:** Department of Mechanical Engineering, Ulsan National Institute of Science and Technology (UNIST), 50 UNIST-gil, Eonyang-eup, Ulju-gun, Ulsan 44919, Korea; gmcljw@unist.ac.kr (J.L.); rnfnr225@gm.gist.ac.kr (S.K.)

**Keywords:** heavy metal sensor, hierarchical nanoporous carbon, sponge-like edge, sensitivity, C-MEMS

## Abstract

This article presents the development of a highly sensitive electrochemical heavy metal sensor based on hierarchical porous carbon electrodes with sponge-like edge structures. Micrometer-scale hierarchical nanoporous carbon electrodes were fabricated at a wafer-scale using cost-effective batch microfabrication technologies, including the carbon microelectromechanical systems technology and oxygen plasma etching. The sponge-like hierarchical porous structure and sub-micrometer edges of the nanoporous carbon electrodes facilitate fast electron transfer rate and large active sites, leading to the efficient formation of dense heavy metal alloy particles of small sizes during the preconcentration step. This enhanced the peak current response during the square wave anodic stripping voltammetry, enabling the detection of Cd(II) and Pb(II) at concentrations as low as 0.41 and 0.7 μg L^−1^, respectively, with high sensitivity per unit sensing area (Cd: 109.45 nA μg^−1^ L mm^−2^, Pb: 100.37 nA μg^−1^ L mm^−2^).

## 1. Introduction

Heavy metal ions can accumulate in the human body and cause serious damage to organs. For example, cadmium and lead cause disorders of the nervous system, kidney, liver, and bones, even at a small quantity [1]. The standard guidelines of the US Environmental Protection Agency (EPA) restrict the concentration of cadmium and lead in drinking water at 5 and 15 ppb, respectively [2]. The well-developed heavy metal analysis methods based on spectroscopy, such as inductively coupled plasma mass spectrometry, atomic absorption spectroscopy, and X-ray fluorescence (XRF) spectroscopy [3,4,5], can facilitate the detection of heavy metals at concentrations as low as parts per trillion in a laboratory [6]. However, these laboratory techniques require long analysis times and skilled personnel [7,8]. In addition, field detection is limited because of the bulky equipment size. Although portable XRF spectrometers are relatively free from these limitations, their limit of detection (LOD) is not sufficient for detecting heavy metal ions at parts per billion levels [9].

Owing to the simple configuration and small size, electrochemical and colorimetric analysis methods have been adopted for on-site heavy metal detection. In addition, these methods facilitate a more sensitive detection of multiple heavy metal ions at a lower cost, as compared to that of the portable XRF spectrometry [10,11,12]. Recently, Ying Gan et al. reported the successful detection of cadmium at concentrations as low as 1.12 ppb, using a colorimetric method; this concentration is much lower than the EPA standard for cadmium concentration in drinking water [13]. However, the colorimetric method still has limitations, such as a relatively complex sample preparation step and low sensitivity in the detection of multiple heavy metal ions, when compared to the electrochemical method [14]. Square wave anodic stripping voltammetry (SWASV) has been widely used for the electrochemical detection of heavy metals because multiple heavy metals can be distinguished with high sensitivity, according to their characteristic oxidation potential [15,16]. SWASV consists of two steps: (i) a preconcentration step in which the heavy metal ions in the analyte are concentrated by electrodeposition and (ii) an anodic removal step in which the electrodeposited metal alloy is oxidized to generate a peak current signal for each metal.

In early studies, mercury was employed as the electrode material for the anodic stripping method-based heavy metal sensors because it facilitates efficient amalgam formation, leading to high sensitivity and good reproducibility [17,18,19]. However, the toxicity of the mercury has limited its further application in heavy metal sensor electrodes, and bismuth has consequently received attention as a non-toxic electrode material [20,21]. For an electrochemical electrode of a heavy metal sensor, a nanoporous structure is generally used to enlarge the adsorption sites and improve the diffusion and local concentration of the target ions [22,23]. In particular, carbon-based porous electrodes are widely used in the bismuth-modified heavy metal sensors because of their good conductivity among the porous electrode materials, wide potential window, and low cost [20,21,24]. In addition, when the porous electrode has a hierarchical architecture, the electrode provides excellent advantages. For example, the macropores and mesopores enhance the diffusion of the electrochemical species into the nanopores; the adsorption sites are increased, facilitating excellent physical and chemical adsorption of metal ions; and the reaction kinetics is improved due to the small diffusion length in the 3D porous structure [25,26,27]. Previously, hierarchical nanoporous carbon electrodes fabricated by eco-friendly methods using biomass were developed to improve the heavy metal adsorption [25,26,28]. The biomass-induced nanoporous electrode-based heavy metal sensor allowed the sensitive detection of heavy metal ions at low concentrations.

In this study, we developed a highly sensitive heavy metal sensor based on patternable hierarchical nanoporous carbon electrode. The disk-shaped carbon electrode with hierarchical sponge-like porous networks was prepared by the fabrication technology of carbon microelectromechanical systems (C-MEMS) and microwave O_2_ plasma etching, which facilitated 6-inch wafer-scale batch fabrication, ensuring cost-effective sensor production. C-MEMS is capable of generating glassy carbon micro/nanostructures by pyrolyzing photosensitive polymer structures pre-patterned by using various lithography technologies, including photolithography and e-beam lithography [29,30]. The C-MEMS-based glassy carbon is a good electrode material for electrochemical analysis, owing to its advantageous properties, such as high thermal stability, excellent electrochemical stability, and a wide potential window [31]. For this reason, the glassy carbon microelectrodes have been widely used in a variety of electrochemical analysis applications, such as biomolecule detection [32,33], bacterial sensing [34], cell sensing [35], and heavy metal detection [36]. In addition, the glassy carbon has good corrosion resistance, allowing the heavy metal sensor to operate stably during the preconcentration and stripping steps in SWAV. In addition to the patternability of the C-MEMS-based microelectrode, nanowire forest-like porous surface can be formed in a facile manner, by treating polymer precursors, using O_2_ plasma prior to pyrolysis [37]. In this study, a hierarchical sponge-like porous networks could be formed via microwave O_2_ plasma, which generates high plasma density with low ion bombardment energy [38]. The sponge-like edge structure, as well as the large surface area derived from the hierarchical nanostructure, enabled efficient and conformal electrodeposition of the metals in the preconcentration step, leading to high sensitivity in the simultaneous detection of dual heavy metal ions, Cd(II) and Pb(II). In addition, the hierarchical nanoporous electrode exhibited a large current density (peak current/unit sensing area), facilitating heavy metal detection with a low LOD, even with a micrometer sized electrode. In this study, the effect of the hierarchical porous architecture on the heavy metal sensing was analyzed by comparing the preconcentration behaviors and the corresponding anodic stripping current signals of Cd and Pb ions, using carbon electrodes with different surface porosities. The reliability of the patternable hierarchical nanoporous carbon electrode-based heavy metal sensor was verified by using tap water spiked with heavy metal ions.

## 2. Materials and Methods

### 2.1. Materials

A negative photoresist (SU-8 2025, Kayaku Advanced Materials, Inc., Westborough, MA, USA) was used as the precursor for the pyrolyzed carbon electrode, while a positive photoresist (AZ 40 XT, Merk KGaA, Darmstadt, Germany) was used as the mask material for the insulation layer patterning. Potassium ferrocyanide (K_4_Fe(CN)_6_), potassium chloride (KCl), potassium sulfate (K_2_SO_4_), and standard solutions of Cd and Pb were purchased from Sigma-Aldrich (St. Louis, MO, USA). The standard solution of bismuth was purchased from Kanto Chemical Co., Inc. (Tokyo, Japan). Acetic acid (glacial, 99.7%) was purchased from Samchun Chemical Co. (Seoul, Korea). Sodium acetate trihydrate was purchased from Junsei Chemical Co., Ltd. (Tokyo, Japan). The buffered oxide etch (BOE) solution (JT Baker) was purchased from Fisher scientific (Waltham, MA, USA). Tap water was obtained from waterworks in Ulsan, South Korea.

### 2.2. Fabrication of the Hierarchical Nanoporous Carbon Electrode with Sponge-Like Edge Structures

**Figure 1:**
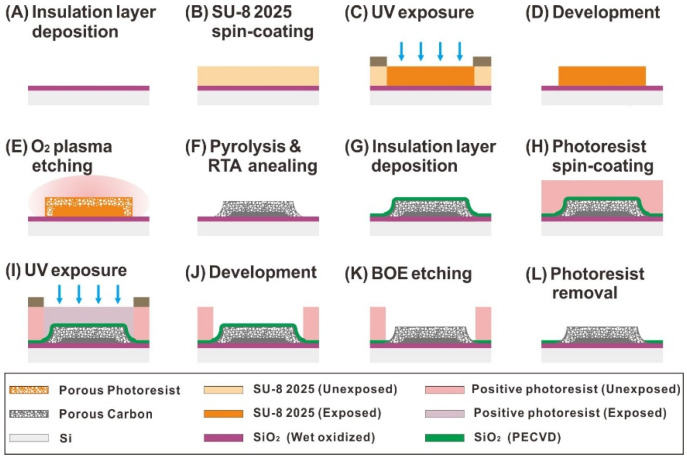
Schematic of the fabrication steps for hierarchical nanoporous carbon electrodes (PR, photoresist; RTA, rapid thermal annealing; BOE, buffered oxide etch).

Hierarchical nanoporous-carbon-structure-based sensors of heavy metals were fabricated in a controllable manner through C-MEMS and O_2_ microwave plasma etching. The dimensions and geometries of the fabricated structures were inspected, using a surface profiler (P6, KLA Corporation, Milpitas, CA, USA) and a scanning electron microscope (Quanta200 FE-SEM, FEI Company, Hillsboro, OR, USA). First, 25 μm thick photoresist structures were patterned using the SU-8 2025 negative photoresist by UV-lithography on top of a thermally oxidized 6-inch silicon wafer, as shown in Figure 1A–D. Then, the surface of the photoresist was subjected to O_2_ microwave plasma etching (V15-G, KAMI, Linden, Germany), to controllably form a structure with surface porosity and sponge-like networks, as shown in Figure 1E. In this study, the surface porosity was controlled by changing the microwave plasma etching time, while the flow rate (O_2_ 100 sccm), plasma power (500 W), and chamber pressure (60 mTorr) were fixed. After the oxygen-plasma-etching process, the photoresist structures were pyrolyzed in a vacuum furnace (DMTF15/145-400, Daemyoung Enterprise Co., Ltd., Gwangmyeong-si, Korea), to convert the polymer structures into carbon electrodes, while maintaining the surface porosity. To enhance the electrical conductivity, the pyrolyzed porous carbon electrodes were subsequently subjected to a rapid thermal-annealing process (KVR-6000, Korea Vacuum Tech., Ltd., Gimpo-si, Korea) at 1000 °C, as described in our previous report [39]. The nanoporous carbon electrodes were finally insulated with a silicon dioxide layer via plasma-enhanced chemical vapor deposition (PEH-600, SORONA Inc., Anseong-si, Korea), as shown in Figure 1G. The active sensing area of the nanoporous carbon electrodes was exposed through UV lithography and BOE wet-etching processes, as shown in Figure 1H–L. 

### 2.3. Electrochemical Characterization of Hierarchical Nanoporous Carbon Electrodes

The electrochemical properties of the prepared nanoporous carbon electrodes with different surface morphologies were characterized by cyclic voltammetry (CV) and SWASV. A three-electrode system was used for all the electrochemical experiments; a commercial Ag/AgCl reference electrode (RE-1B, ALS Co., Ltd., Tokyo, Japan), a platinum wire, and the prepared nanoporous carbon electrodes were used as the reference, counter, and working electrode, respectively. The surface area of the electrode was determined electrochemically through CV in a 0.2 M K_2_SO_4_ solution, by scanning the working electrode from 0 to 0.8 V vs. an Ag/AgCl electrode. The electrochemical reactivity of the electrode surface was characterized, using CV of 10 mM K_4_Fe(CN)_6_ in 0.5 M KCl. The heavy metal alloy of bismuth, cadmium, and lead was electrodeposited at −1.4 V, to analyze the effect of the morphology of the electrode surface on the preconcentration behaviors. These electrochemical characterizations were performed, using a multi-potentiostat (CHI 1020, CH Instruments, Inc., Austin, TX, USA). The heavy-metal-detection capability of nanoporous carbon electrodes was evaluated by SWASV, using a potentiostat (Modulab XM MTS, Solatron Analytical, Hampshire, UK). Bismuth and the target heavy metals were electrodeposited in the form of an alloy on the nanoporous carbon electrodes at a reduction potential of −1.4 V vs. an Ag/AgCl electrode for 300 s. Then, the electrodeposited alloy was stripped with a square wave of 15 Hz frequency, 25 mV pulse amplitude, and 5 mV step potential; this resulted in anodic stripping current signals. The nanoporous carbon electrodes were electrochemically cleaned by applying 0.2 V vs. Ag/AgCl reference electrode for 60 s in 0.1 M NaAc buffer solution between each measurement. Tap water from Ulsan, South Korea, was used to validate the heavy metal sensor in the analysis of real samples. The tap water was spiked with Cd and Pb ions, and the solution was diluted with 0.1 M NaAc buffer at a 1:1 ratio. The composition of the carbon electrodes, according to O_2_-plasma-etching time, was analyzed by using X-ray photoelectron spectroscopy (XPS; ESCALAB 250XI, Thermo Fisher Scientific Inc., Waltham, MA, USA). The compositions of tap-water samples were analyzed by ICP-MS (Agilent 7700s, Agilent Technologies, Inc., Santa Clara, CA, USA).

## 3. Results and Discussion

### 3.1. Morphology of the Nanoporous Carbon Electrode with a Sponge-Like Edge Structure

**Figure 2:**
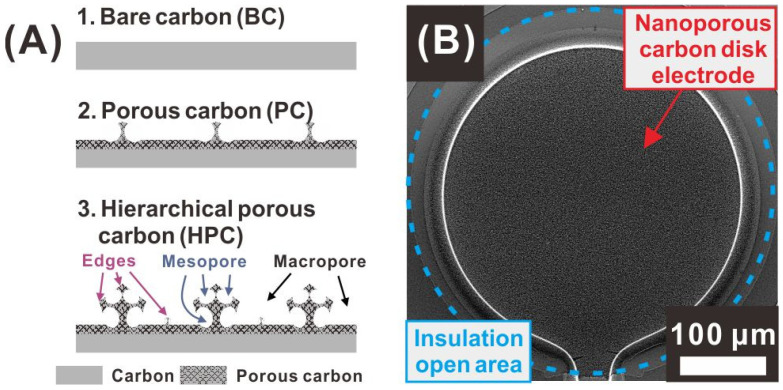
(**A**) Schematic sectional views of nanoporous carbon electrodes, according to their porosity. (**B**) Scanning electron microscopy image of a hierarchical porous carbon (HPC) disk electrode.

Three types of carbon electrodes with different porosities and surface morphologies are categorized as bare carbon (BC), porous carbon (PC), and hierarchical porous carbon (HPC), based on the surface porosity achieved depending on the O_2_-plasma-etching time (Figure 2A). In this study, microwave plasma was used to form nanoporous sponge-like networks on the surface of SU-8 photoresist structures. This negative photoresist polymer contains antimony ions that have a high etching resistance to O_2_ plasma, and these ions act as etch masks. In addition to this self-mask material, the difference between the etching rates of the aromatic and aliphatic parts of the photoresist also causes the formation of a nanoporous surface [37,40]. Furthermore, the microwave plasma-etching process generates etching species with low ion bombardment energy, leading to isotropic etching. Therefore, sponge-like nanoporous networks consisting of pores and narrow edge structures are formed instead of vertically aligned nanostructures, as illustrated in the sectional schematics of the PC and HPC electrodes (Figure 2A). As the plasma-etching proceeds, the polymer surface is removed progressively, and, thus, the top macropore size increases and additional nanoporous layers are formed below the top macropores, leading to the formation of sponge-like hierarchical porous networks, as shown in the sectional view of the HPC electrode in Figure 2A. The HPC, PC, and BC electrodes were prepared in the form of disks of 400 μm diameter, as shown in Figure 2B.

**Figure 3:**
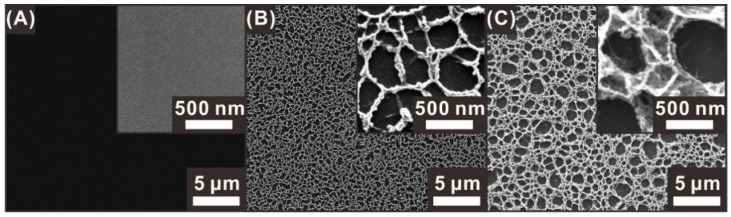
SEM images of carbon electrodes with different surface porosities formed depending on the O_2_-plasma-etching time: (**A**) bare carbon (BC) electrode (no plasma etching process), (**B**) porous carbon (PC) electrode (300 s plasma etching), and (**C**) HPC electrode (900 s plasma etching).

The surface morphology of each electrode was observed by scanning electron microscopy (SEM). Before the O_2_-plasma-polymer-etching process, the pyrolyzed carbon electrode (BC electrode) exhibited a smooth surface without any micro/nanostructures, as shown in Figure 3A. The PC electrode obtained by pyrolyzing a negative photoresist structure treated with an O_2_ plasma process for 300 s exhibited nanoscale edge networks, with micrometer-sized cavities, as shown in Figure 3B. A longer O_2_-plasma-etching process (900 s) resulted in the HPC electrode with a 3D sponge-like hierarchical nanoporous network, as shown in Figure 3C. This HPC electrode exhibited the largest cavity (50 nm to 2 μm) at the top surface and additional smaller porous networks formed below the pre-formed larger pore structures, leading to a higher surface area, compared to those of the PC and BC electrodes, as demonstrated in the ensuing section.

### 3.2. Effect of the Electrode Surface Properties on the Heavy Metal Detection

**Figure 4:**
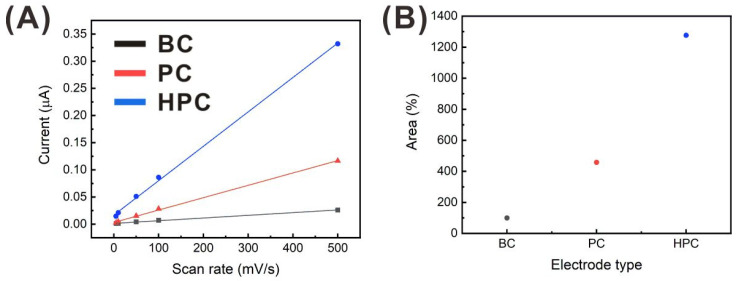
(**A**) Capacitive current difference at the middle of the potential window, according to the scan rate, and (**B**) the corresponding electrochemically active surface area (EASA) ratios of the HPC and PC electrodes, compared to that of the BC electrode.

The effect of the plasma-etching time-dependent nanoporous morphology on the electrochemically active surface area (EASA) of the carbon electrodes was evaluated by using their double-layer capacitances calculated from CVs, as described by the following:

EASA = C_dl_/C_s_ (1)

where C_dl_ is the double-layer capacitance, and *C_s_* is the specific capacitance of the standard materials [41,42]. First, the CVs were collected from a 0.2 M potassium sulfate solution at various scan rates, as shown in Supplementary Materials Figure S1. Then, the double-layer capacitance, which corresponds to the slope of the linear regression curve of the difference between the currents at the middle of the potential window ((i_anodic_, 0.4 V – i_cathodic_, 0.4 V)/2) versus the scan rate (Figure 4A), was determined. The specific capacitance of the standard materials was assumed to be the same for all electrodes. Thus, the relative ratios of the EASA of the nanoporous electrodes (PC and HPC), compared to that of the BC electrode were estimated (Figure 4B), according to the linear relationship between the EASA and double-layer capacitance. The PC and HPC electrodes exhibited 4.6 and 12.8 times greater EASA than that of the BC electrode, respectively. As mentioned earlier, the hierarchical porous networks were well-developed in the HPC electrode, leading to the largest surface area.

**Figure 5:**
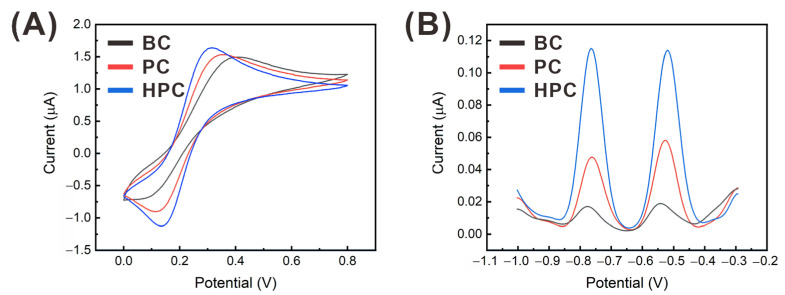
(**A**) Cyclic voltammograms of 10 mM ferrocyanide in 0.5 M KCl at the scan rate of 0.05 V/s. (**B**) Square wave anodic stripping voltammetry (SWASV) current signals from 10 μg L^−1^ of each of cadmium and lead, along with 400 μg L^−1^ of bismuth in a NaAc buffer measured by using the BC, PC, and HPC electrodes.

The electrochemical properties of the carbon electrodes were characterized by using CV of 10 mM ferrocyanide in 0.5 M KCl, as shown in Figure 5A. As the porosity increased, the peak-to-peak separation (ΔE_p_) decreased, indicating enhanced surface reactivity. However, the diffusion-limited current did not change significantly and even decreased with the porosity, although the EASA values of the nanoporous electrodes were significantly higher than that of the BC electrode. This is because most of the electrochemical species are consumed at the top of the nanoporous surface, owing to the efficient electron transfer rate, and the deeper pore regions far from the bulk solution do not participate in the redox reaction [43]. In addition, it should be emphasized that the thickness of the carbon electrodes decreased with the O_2_-plasma-etching time (thickness: BC, 4.5 μm; PC, 3.7 μm; HPC, 3.4 μm), and, thus, the overall surface area was reduced.

The effect of the surface porosity of the carbon electrode on the SWASV-based heavy metal detection was evaluated with 10 μg L^−1^ of each of cadmium and lead, and 400 μg L^−1^ of bismuth in a NaAc buffer solution. The stripping peak currents were observed between −0.7 and −0.8 V for cadmium and −0.45 to −0.6 for lead, and the current signals increased with increasing the porosity, as shown in Figure 5B. Compared to the increase in the electrochemical performance with the porosity demonstrated by the CV tests, the SWASV peak current was more significantly enhanced. For further analyzing the reason for the enhanced sensing performance of the HPC electrode, the preconcentration behaviors were investigated according to the surface morphology. 

**Figure 6:**
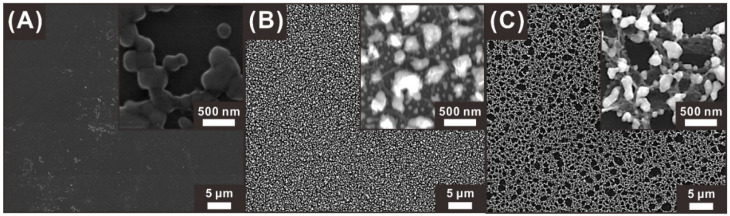
SEM images of (**A**) BC, (**B**) PC, and (**C**) HPC electrodes with the electrodeposited heavy metal alloys (Cd, 10 mg L^−1^; Pb, 10 mg L^−1^; Bi, 400 μg L^−1^).

In the preconcentration step, the deposition behavior of the heavy metal ions is affected by the surface morphology of the electrode, and this behavior strongly affects the anodic current signals in the detection of heavy metal ions. Thus, the effect of the porous surface morphology on the heavy metal detection was investigated by studying the electrodeposition behavior of the alloys of cadmium and lead with bismuth on the three carbon electrodes (BC, PC, and HPC). For a clear observation of the heavy metal alloy, excessive amounts of cadmium (10 mg L^−1^) and lead (10 mg L^−1^) were electrodeposited on each type of the electrode for 30 min (–1.4 V vs. Ag/AgCl electrode), without an anodic stripping step. The bismuth concentration was fixed at the heavy metal detection condition (400 μg L^−1^ in the NaAc buffer). The heavy metal alloy deposition differed in shape and distribution, depending on the surface porosity of the electrodes, as shown in SEM images and corresponding energy-dispersive X-ray spectroscopy (EDS) mapping. The BC electrode exhibited relatively large heavy metal alloy islands that were irregularly distributed at a low density (Figure 6A and Supplementary Materials Figure S2). The EDS mapping exhibited a negligible amount of Bi, because excessive amount of Pb and Cd were deposited as mentioned above. In contrast, metal alloy particles were deposited at a significantly higher density on the PC electrode surface. Specifically, the metal particles were mainly located on the edges of the macropores, as shown in Figure 6B and Supplementary Materials Figure S3. The HPC also exhibited similar behavior as that of the PC electrode, but the metal particles were distributed deeper in the hierarchical nanoporous network with smaller particle sizes than those on the PC electrode (Figure 6C and Supplementary Materials Figure S4). During the electrodeposition, protruded features of the electrode function as more electrochemically active sites, owing to the enhanced local electric field, leading to more efficient deposition on them than on the planar electrode region [22,23]. Therefore, metal particles were deposited along the sponge-like edge networks of the nanoporous carbon electrode surface, resulting in conformal and dense particle deposits. In addition, as the porosity increased, the particle size decreased due to the enlarged active sites under the fixed metal ion concentration condition. Therefore, this conformal and dense deposition of heavy metal alloy particles enhanced the anodic-stripping current signal. Moreover, the smaller metal particles formed on the HPC electrode facilitated more efficient anodic stripping, owing to the larger surface-to-volume ratio of the particle. 

The XPS measurements were carried out to analyze the composition change of the pyrolyzed carbon electrodes, according to O_2_-plasma-etching time. In the C1s region, no significant difference in peak intensity at 283.7 eV, which represents aromatic and aliphatic carbons, was shown between BC, PC, and HPC electrodes (Supplementary Materials Figure S5A). In contrast, the PC and HPC electrodes show higher peak intensities compared to the BC electrode in the O1s spectra region (Supplementary Materials Figure S5B). Therefore, the atomic ratio of O/C increases with the oxygen-plasma-etching time. The reason for this can be inferred from the previous study on the oxygen-plasma-treated SU-8, using XPS by Lim et al. [38]: As the O_2_ plasma etching proceeds, the atomic ratio of O/C increases due to the partial oxidation on the SU-8 surface [44]. Thus, the enhancement of the adsorption of the heavy metal ions in the oxygen-plasma-treated carbon can be attributed to the evolved oxygen-containing functional groups [26].

In conclusion, the increased EASA of the hierarchical nanoporous electrode did not significantly enhance the Faradaic reaction, but the well-developed networks of sponge-like edges and enhanced oxygen functional group facilitated the formation of dense small metal alloy particles, leading to enhanced heavy-metal-detection capability.

### 3.3. Optimization of the Preconcentration Conditions

As stated in the previous section, the HPC exhibited the best heavy-metal-sensing capability, owing to its enhanced surface reactivity and adsorption capability. Therefore, the HPC electrode was used for further heavy-metal-sensing studies. First, the effects of preconcentration conditions, including the pH, potential, duration, and Bi concentration on the anodic stripping current signal, were analyzed, using the HPC electrode. The maximum stripping current response was obtained at pH 4.5 for both the cadmium and lead ions, as shown in Supplementary Materials Figure S6A. The peak current response increased as the HPC electrode was biased at higher magnitudes of the negative potential and decreased beyond −1.4 V, because of the adverse effect of hydrogen evolution (Supplementary Materials Figure S6B). With the pH and potential fixed at 4.5 and –1.4 V, respectively, the duration and Bi concentration of the preconcentration step were optimized. The current signals corresponding to both the ions, cadmium and lead, increased continuously with increasing preconcentration time (until 300 s) and Bi concentration (up to 400 μg L^−1^), and then saturated (Supplementary Materials Figure S6C,D). Therefore, these conditions were selected for heavy metal sensing.

### 3.4. Simultaneous Detection of Cadmium and Lead, Using the HPC Electrode

**Figure 7:**
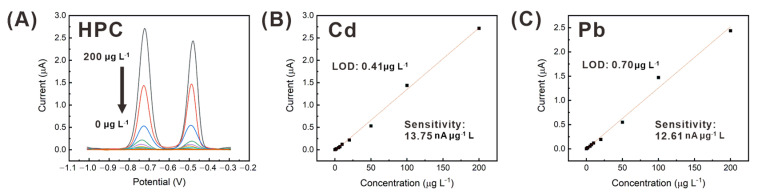
(**A**) SWASV plots obtained at various concentrations (0, 1, 2, 5, 7, 10, 20, 50, 100, and 200 μg L^−1^) of cadmium and lead in a NaAc buffer, and peak current signals at various concentrations of (**B**) cadmium and (**C**) lead measured by using a 400 μm diameter disk HPC electrode.

Based on the in situ formation of the bismuth alloy and the acquisition of the current signal by SWASV, cadmium and lead were detected simultaneously at various concentrations (0–200 μg L^−1^), using HPC electrodes. Distinct peak currents in the ranges of −0.7 to −0.8 V and −0.45 to −0.55 V were obtained from cadmium and lead, respectively, as shown in Figure 7A. A shift in the peak potential was observed upon varying the heavy metal concentrations. Nevertheless, the peak potentials from cadmium and lead in the concentration range of 0 to 200 μg L^−1^ could be clearly distinguished. Further, the peak current signals from the SWASV results (Figure 7A) were plotted as a function of the heavy metal concentration to analyze the performance of the HPC electrode-based heavy metal sensors, as represented in Figure 7B,C. The sensor showed a linear relationship between the peak current signal and concentration over a wide range of the heavy metal concentration (0–200 μg L^−1^). Thus, the reliability of the sensor in the detection of cadmium and lead was evaluated by using a linear regression line. The HPC electrode showed a linear correlation with good R^2^ value (cadmium: 0.996, lead: 0.990) over the entire concentration range, facilitating excellent LOD (cadmium: 0.41 μg L^−1^, lead: 0.7 μg L^−1^, S/N = 3). The LOD values were calculated based on the linear regression line of the current signals at various heavy metal concentrations, as described in Equation (2):

LOD = a + 3S_a_ (2)

where a is the y-intercept, and S_a_ is the standard deviation of the y-intercept [45]. These LOD values are much lower than the EPA standard for drinking water (cadmium: 5 μg L^−1^, lead: 15 μg L^−1^) [2]. Such detection of cadmium and lead at very low concentrations, using the small-sized electrode (0.1256 mm^2^), was enabled by the high sensitivity per unit sensing area (cadmium: 109.45 nA μg^−1^ L mm^−2^, lead: 100.37 nA μg^−1^ L mm^−2^), owing to the good surface reactivity and large active site area of the hierarchical sponge-like edge structures.

**Figure 8:**
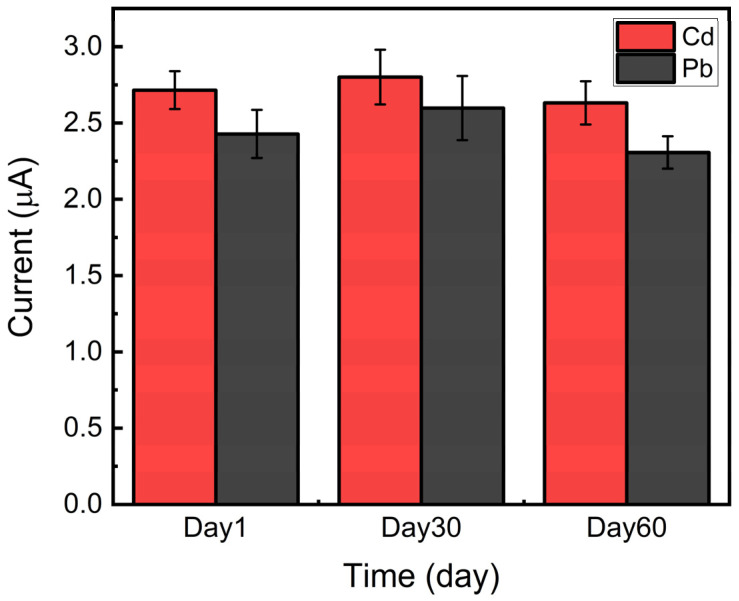
Reproducibility and stability test, using five different HPC electrodes for a two-month period (red bar, 200 ppb Cd; black bar, 200 ppb Pb).

To investigate the applicability of the present heavy metal sensor based on HPC to practical applications, the reproducibility and long-term stability were tested. Five different HPC electrodes were prepared, and their electrochemical current signals of 200 ppb of Cd and Pb were measured once a month over two months. As shown in Figure 8, the sensors exhibited a relatively good reproducibility of 7.8% RSD, when comparing the sensor signals between sensors. In addition, the current signal from sensors exhibited a slight deviation of 6.6% RSD when the sensors were reused after each one-month storage at atmospheric conditions.

**Figure 9:**
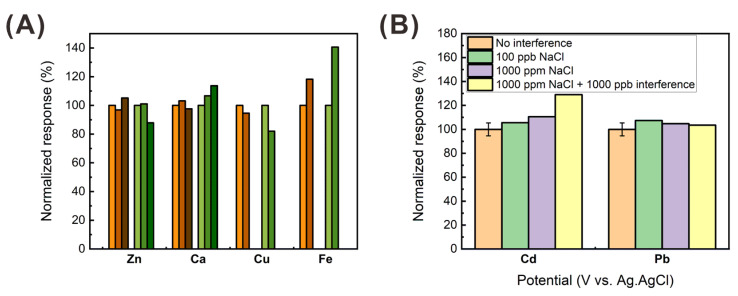
Interference effect on the heavy metal detection of the HPC-based heavy metal sensor: (**A**) normalized peak current signals of 20 ppb Cd (brown color) and Pb (green color) against metal ion interferences (Zn, Ca, Cu, and Fe) of 100 and 20 ppm; (**B**) normalized peak current signals of 20 ppb Cd (left bars) and Pb (right bars) against NaCl and NaCl with metal ion interferences (Zn, Ca, Cu, and Fe).

The selectivity of the HPC-based heavy metal sensor was evaluated in the presence of other potentially interfering metal ions and inorganic anions, such as Cu, Zn, Ca, Fe, Na, and Cl. Tap water in Ulsan, South Korea, was reported to contain negligible amounts of Cd, Pb, Cu, and Fe, and 41.5 ppm of chloride ion, according to the accredited test result of Korea Laboratory Accreditation Scheme, which was signed by the ILAC-MRA. In addition, we confirmed that negligible amounts of Cd, Pb, Cu, and Fe were contained in the tap-water sample, using ICP-MS, as shown in Supplementary Materials Table S1. Thus, in this study, the peak current signals of 20 ppb cadmium and lead were measured with the presence of 100 ppb Zn, Ca, Cu, and Fe, as shown in Figure 9A. This test was also performed with excessive amounts of Ca and Zn (20 ppm). In the presence of Zn and Ca, the current signals of Cd and Pb changed to less than 3.2 and 6.7%. In contrast, Cu and Fe induced comparably larger signal change. However, this relatively large signal change (Cd: ~18%, Pb: ~40%) corresponds to only 2.9 and 6.2 ppb difference in Cd and Pb detection, respectively, owing to the high sensitivity of the HPC-based sensor. The effect of NaCl on the heavy metal detection was also evaluated by measuring the peak currents of Pb and Cd, in the presence of various concentrations of NaCl and the mixture of 1000 ppm NaCl and 1000 ppb metal ions (Zn, Ca, Cu, Fe), as shown in Figure 9B. The organic anion also exhibited minor change in Cd and Pb detection by 1.7 and 0.7 ppb, respectively. Considering that Cu and Fe are contained in the tap water less than 100 ppb, the presented heavy metal sensor showed acceptable selectivity for the detection of Cd and Pb ions in the presence of the various interfering metal and inorganic ions.

### 3.5. Heavy Metal Detection in a Real Water Sample

**Figure 10:**
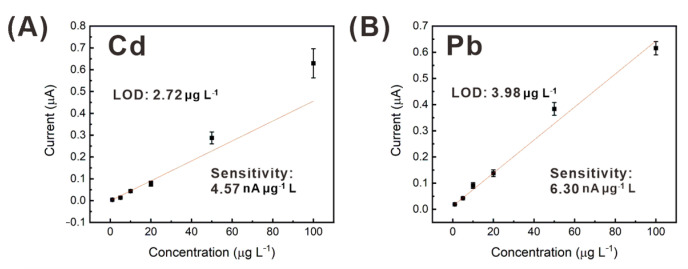
SWASV peak current signals from a real tap-water sample spiked with (**A**) cadmium and (**B**) lead at various concentrations (0, 1, 5, 10, 20, 50, and 100 μg L^−1^), obtained by using the HPC-based heavy metal sensor.

The performance of the HPC-based heavy metal sensor in the analysis of real water sample was evaluated, using tap water. For the comparative correlation study, the real samples with addition of Cd and Pb were analyzed, using both ICP-MS and HPC electrodes. The HPC electrode showed good agreement with the ICP-MS results with relative errors less than 7% as shown in Table 1. The %RSD values of the measurement using the HPC electrode were greater, compared to ICP-MS. However, considering the EPA standard (Cd 5 ppb and Pb 15 ppb), it is considered acceptable for testing tap-water samples. Supplementary Materials Figure S7 shows the SWASV curves of the HPC electrode obtained from a tap-water sample spiked with cadmium and lead at known concentrations. Peak current signals obtained from tap-water samples spiked with 0–100 μg L^−1^ cadmium and lead are shown in Figure 10. For the linear regression, the mean values of the peak currents measured using five different HPC-based sensors were used. The linear fitting curves show relatively good R^2^ values of 0.921 and 0.9757 for cadmium and lead, respectively, but the sensitivity of the HPC-based sensor was lower for the tap water, compared to that for deionized water-based samples (Figure 7B,C). However, the HPC-based sensor exhibited sufficient LOD (cadmium: 2.72 μg L^−1^, lead: 3.98 μg L^−1^) in the analysis of the real tap-water sample, to enable the detection of metal ions, at concentrations as low as the EPA standard for drinking water.

## 4. Conclusions

In this study, a sensitive heavy metal sensor was developed based on a micrometer-scale sponge-like hierarchical nanoporous carbon electrode. The recently developed electrochemical heavy metal sensors have employed various types of nanostructured electrodes with large surface area and excellent surface reactivity, facilitating the detection of heavy metals at low concentrations (0.2~20 ppb), as listed in Table 2. However, these sensors did not exhibit sufficient sensitivity per unit sensing area, to enable stable current signal measurement with a miniaturized sensor. The presented HPC electrode-based heavy metal sensor exhibited high sensitivity and low LOD at a small electrode size. This was facilitated by the sponge-like edge structure, which allows conformal and dense preconcentration of the heavy metal alloy, in addition to the large surface area and efficient electron transfer rate of the electrode. Thus, the HPC electrode showed superior sensitivity per unit sensing area (Cd, 109.45 nA μg^−1^ L mm^−2^; Pb, 100.37 nA μg^−1^ L mm^−2^) to those of the other nanoporous electrode-based heavy metal sensors (Table 2). Furthermore, the LOD (Cd: 2.72 μg L^−1^, Pb: 3.98 μg L^−1^) and sensing range (0.5–200 μg L^−1^) of the present heavy metal sensor with a micrometer-sized electrode are comparable to those of other heavy metal sensors, satisfying the EPA standard for drinking water. Moreover, HPC electrodes were successfully fabricated at a wafer-scale, using only batch microfabrication methods including C-MEMS and O_2_ plasma etching. This simple manufacturing process is compatible with other MEMS fabrication technologies that facilitate the integration of counter and reference electrodes for the cost-effective production of miniaturized heavy metal sensors.

## Figures and Tables

**Table 1:** Cd and Pb concentrations in tap-water samples spiked with standard heavy metal solutions measured, using ICP-MS and HPC electrodes.

ICP-MS				HPC Electrode				Relative Error (%)
Analyte	Added (μg L^−1^)	Found (μg L^−1^)	Recovery	Analyte	Added (μg L^−1^)	Found (μg L^−1^)	Recovery	
Cd	5	6.11 ± 0.14	122.2	Cd	5	5.70 ± 0.68	114	6.7
Pb	5	4.53 ± 0.19	90.6	Pb	5	4.26 ± 0.20	85.2	6.0
Cd	50	51.94 ± 0.19	103.9	Cd	50	48.36 ± 4.59	96.7	6.9
Pb	50	46.09 ± 0.173	92.2	Pb	50	58.42 ± 3.68	116.8	6.3

**Table 2:** Summary of the sensing performances of various heavy metal sensors, using SWASV method.

Electrode Material	Heavy Metal	LOD (ppb)	Sensing Range (ppb)	* Sensitivity per Unit Sensing Area (nA ppb^−1^ mm^−2^)	Electrode Size (mm^2^)	Real Sample	Reference
(Fe_3_O_4_/TA)NPs modified GCE	Cd	22.48	44.96–123.7	5.508	19.625	River water	[46]
	Pb	8.29	82.88–227.9	2.105			
TiO_2_ nanocrystals modified GCE	Cd	-	22.48–112.4	21.646	-	River water	[47]
	Pb	-	41.44–207.2	20.131			
rGO/CNT/ Bismuth composite	Cd	0.6	20–200	2.62	1.5	Drinking water	[48]
	Pb	0.2	20–200	9.26			
SWCNHs modified SPE	Cd	0.2	1–60	0.114	7	Milk, Honey	[49]
	Pb	0.4	1–60	0.041			
AuNPs/BiNPsmodified CE	Cd	1.51	100–300	18.338	10	Solution in cup	[15]
	Pb	2.20	50–300	15.31			
Functionalizedgold electrode	Pb	0.315	1–400	-	12.56	Tap water	[50]
Kelp-derived porous carbon electrode	Cd	2.62	1.12–56.2	33.37	7.065	Tap water	[28]
	Pb	2.36	2.07–103.6	36.48	7.065		
HPC	Cd	0.41	0.5–200	109.45	0.1256	Tap water	This work
	Pb	0.7	0.5–200	100.37			

* The sensitivity per unit sensing area of each sensor was calculated by dividing the slope of the linear regression line of the detection result by the electrode area. TA, terephthalic acid; NPs, nanoparticles; GCE, glassy carbon electrode; rGO, reduced graphene oxide; CNT, carbon nano tube; SWCNHs, single-walled carbon nanohorns; SPE, screen-printed electrode; CE, carbon electrode; LOD, limit of detection.

## Data Availability

The data presented in this study are available on request from the corresponding author.

## Supplementary Materials

The following are available online, at https://www.mdpi.com/1424-8220/21/4/1346/s1. Figure S1: Cyclic voltammograms of (A) bare carbon (BC), (B) porous carbon (PC), and (C) hierarchical porous carbon (HPC) electrodes in 0.2 M K_2_SO_4_ at different scan rates. Figure S2: (A) SEM image and (B–D) EDS mapping of a BC electrode with electrodeposited heavy metal alloys (Cd, 10 mg L^−1^; Pb, 10 mg L^−1^; Bi, 400 μg L^−1^). Figure S3: (A) SEM image and (B–D) EDS mapping of a PC electrode with electrodeposited heavy metal alloys (Cd, 10 mg L^−1^; Pb, 10 mg L^−1^; Bi, 400 μg L^−1^). Figure S4: (A) SEM image and (B–D) EDS mapping of an HPC electrode with electrodeposited heavy metal alloys (Cd, 10 mg L^−1^; Pb, 10 mg L^−1^; Bi, 400 μg L^−1^). Figure S5: XPS spectrum of BC, PC, and HPC electrodes in (A) C1s and (B) O1s regions. Figure S6: Anodic stripping peak current responses of 50 μg L^−1^ cadmium (black line) and 50 μg L^−1^ lead (red line) in SWASV under different preconcentration conditions: (A) pH, (B) preconcentration potential, (C) preconcentration time, and (D) Bi concentration. Figure S7: SWASV curves obtained at various concentrations (0, 1, 5, 10, 20, 50, and 100 μg L^−1^) of cadmium and lead spiked in tap water buffered with a NaAc solution, using the HPC-based heavy metal sensor. Table S1: ICP-MS analysis of tap-water samples.
