# Supplementary material for: Hierarchical Porous Carbon Electrodes with Sponge-Like Edge Structures for the Sensitive Electrochemical Detection of Heavy Metals
Source: Sensors (Basel). 2021 Feb 14;21(4):1346. doi: 10.3390/s21041346 (PMC7917916; doi:10.3390/s21041346)
Supplement: Supplementary file 1 [file sensors-21-01346-s001.pdf]

Supporting Information: Hierarchical Porous Carbon Electrodes with Sponge-Like Edge Structures for the Sensitive Electrochemical Detection of Heavy Metals

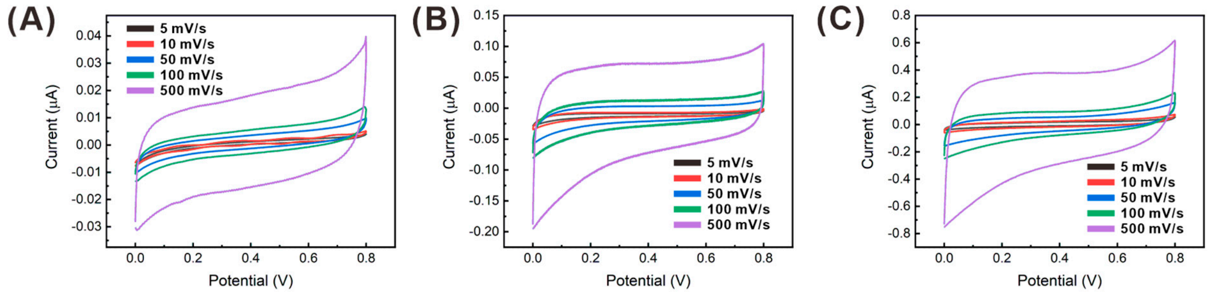

Figure S1. Cyclic voltammograms of (A) bare carbon (BC), (B) porous carbon (PC), and (C) hierarchical porous carbon (HPC) electrodes in 0.2 M K_2SO_4 at different scan rates.

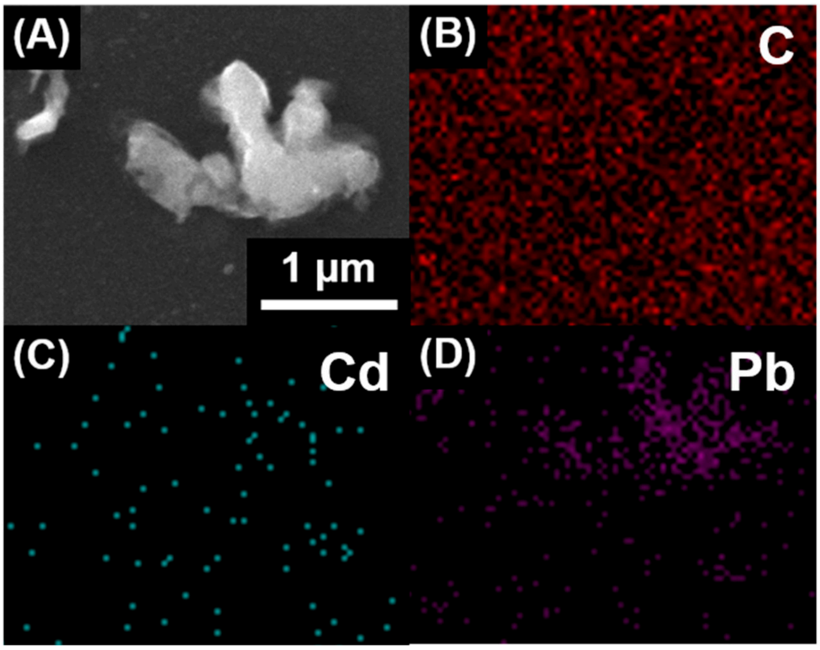

Figure S2. (A) SEM image and (B–D) EDS mapping of a BC electrode with electrodeposited heavy metal alloys (Cd: 10 mg L^{-1}, Pb: 10 mg L^{-1}, Bi: 400 μg L^{-1}).

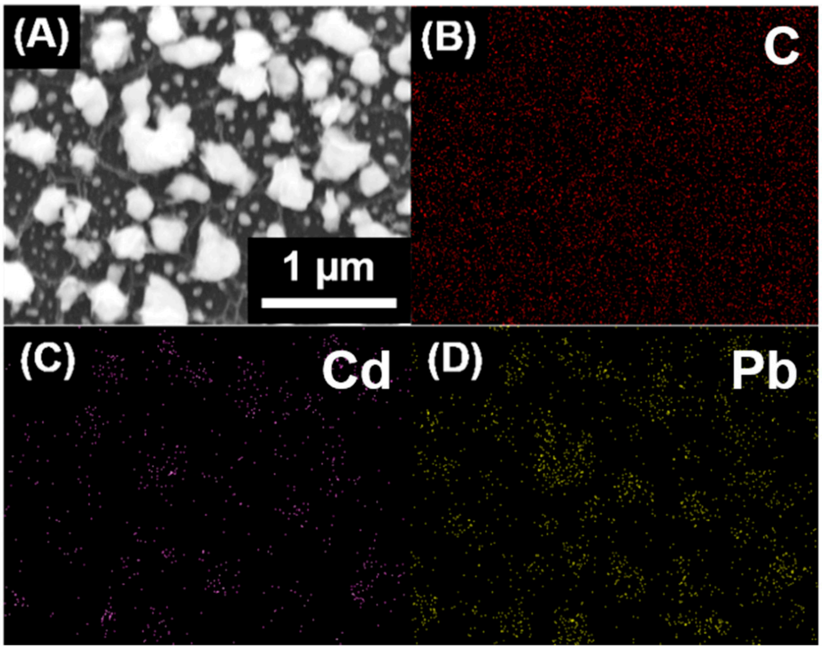

Figure S3. (A) SEM image and (B–D) EDS mapping of a PC electrode with electrodeposited heavy metal alloys (Cd: 10 mg L^{-1}, Pb: 10 mg L^{-1}, Bi: 400 µg L^{-1}).

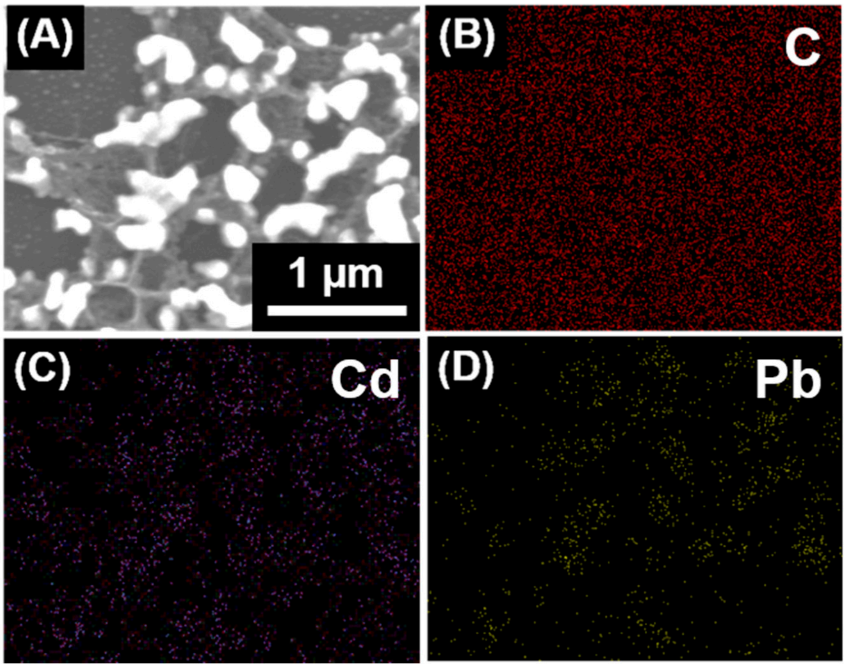

Figure S4. (A) SEM image and (B–D) EDS mapping of a HPC electrode with electrodeposited heavy metal alloys (Cd: 10 mg L^{-1}, Pb: 10 mg L^{-1}, Bi: 400 µg L^{-1}).

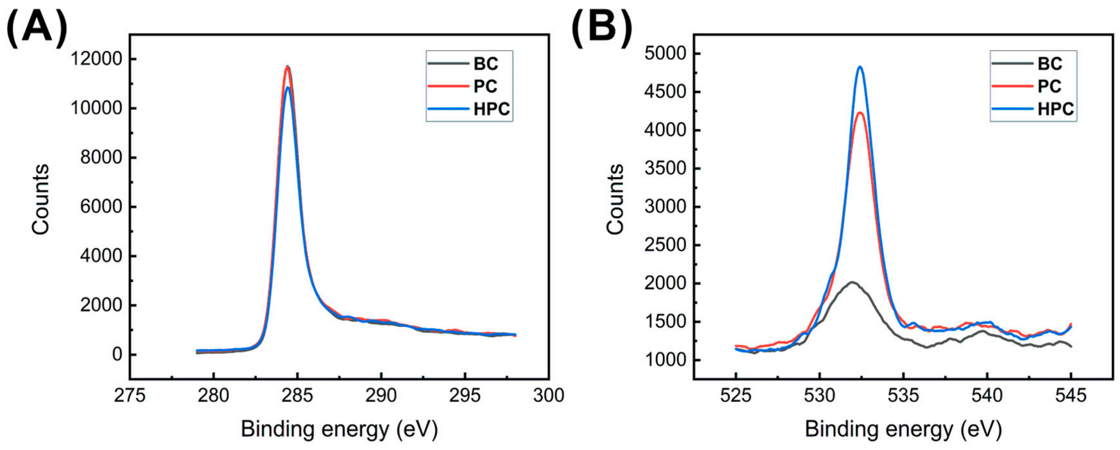

Figure S5. XPS spectrum of BC, PC, and HPC electrodes in (A) C1s and (B) O1s regions.

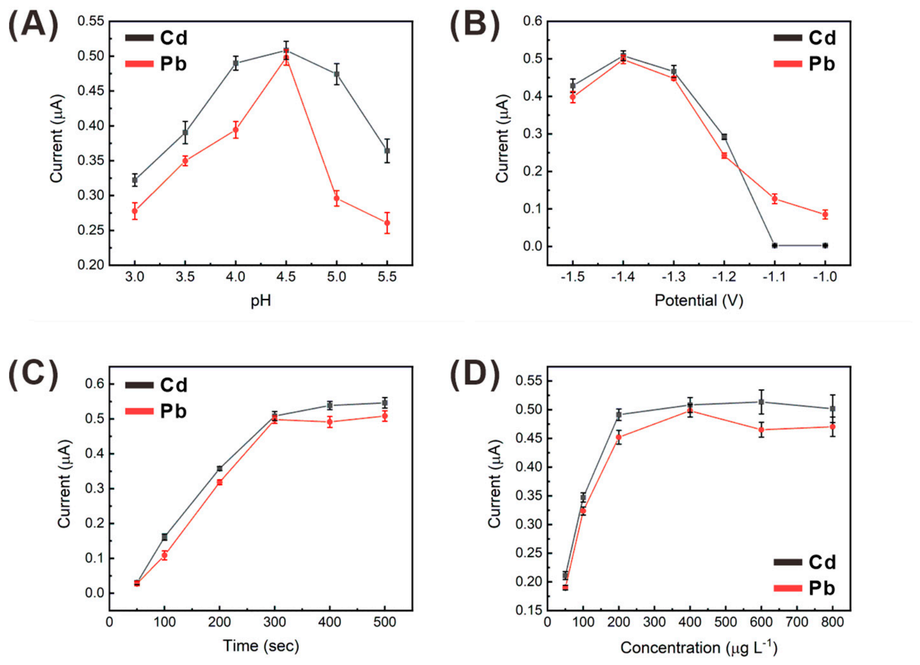

Figure S6. Anodic stripping peak current responses of 50 μg L^{-1} cadmium (black line) and 50 μg L^{-1} lead (red line) in SWASV under different preconcentration conditions: (A) pH, (B) preconcentration potential, (C) preconcentration time, and (D) Bi concentration.

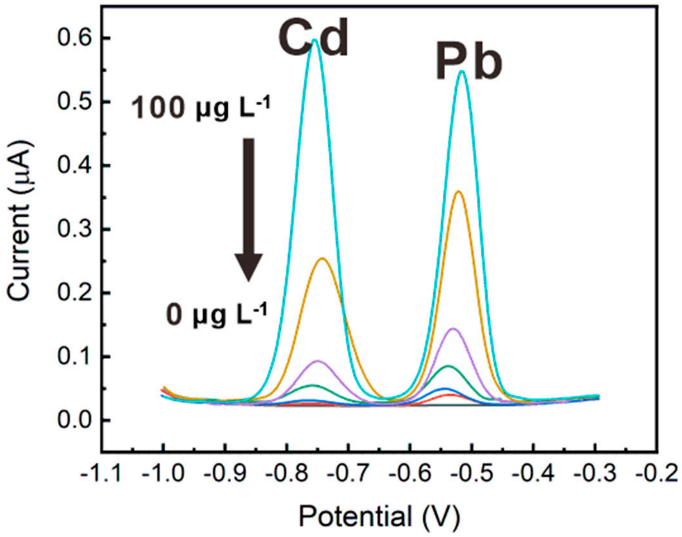

Figure S7. SWASV curves obtained at various concentrations (0, 1, 5, 10, 20, 50, 100 µg L^{-1}) of cadmium and lead spiked in tap water buffered with a NaAc solution using the HPC-based heavy metal sensor.

Table S1. ICP-MS analysis of tap water samples.

Analyte	Found	%RSD	Analyte	Found	%RSD	Analyte	Found	%RSD
Na	15.69 ppm	2.55	Mg	1.24 ppm	4.13	K	11.50 ppm	14.57
Ca	21.34 ppm	12.04	Fe	N/A	-	Cu	6.2 ppb	24.82
Zn	2.77 ppm	12.64	Cd	N/A	-	Pb	N/A	-
